# Autistic Sensory Traits and Psychological Distress: Mediating Role of Worry and Intolerance of Uncertainty

**DOI:** 10.3390/brainsci14111088

**Published:** 2024-10-29

**Authors:** Patricia Recio, Pilar Pozo, Cristina García-López, Encarnación Sarriá

**Affiliations:** 1 Department of Methodology of Behavioral Sciences, Faculty of Psychology, National University for Distance Education (UNED), 28040 Madrid, Spain; reciop@psi.uned.es (P.R.); ppozo@psi.uned.es (P.P.); 2 IMIENS: Joint Research Institute UNED-Health Institute Carlos III, 28029 Madrid, Spain; cristina.garcial@sjd.es; 3 Learning Disabilities Unit (UTAE), Neuropediatrics Department, Hospital Sant Joan de Déu, 08950 Barcelona, Spain; 4 Psychology Department, Faculty of Health Sciences, University Abat Oliba CEU, 08022 Barcelona, Spain

**Keywords:** sensory sensitivities, repetitive behaviors, worry, intolerance of uncertainty, stress, autism, anxiety

## Abstract

Background/Objectives: Autistic adults exhibit core and comorbid features that can have an impact on their daily functioning and lead to considerable psychological distress. Significant and consistent associations have been identified between autism characteristics—restricted repetitive behaviors and sensory features—and high levels of stress and anxiety. For a better understanding of the issue, it is necessary to consider the cognitive aspects that can help explain variations in stress and anxiety levels among adults with autism. We therefore aimed to model the contributions of worry and intolerance of uncertainty on the relationship between autism characteristics (sensory sensitivities and repetitive behaviors) and psychological distress (stress and anxiety). Methods: The sample comprised 144 autistic individuals with a mean age of 35.15 (SD = 11.44). They participated in the study by completing an online questionnaire to assess the study variables. Most of the participants reported being diagnosed with Asperger syndrome (63.6%) or Level 1 autism spectrum disorder (27.1%). Results: The model fit index values tested through path analysis indicated a good fit (χ^2^ = 5.65; *p* = 0.13 ns; CFI = 0.993; NFI = 0.985; RMSEA = 0.079; SRMR = 0.025) and identified worry and intolerance of uncertainty as significant mediating variables within a comprehensive explanatory model. Conclusions: These findings highlight the importance of worry and intolerance of uncertainty as specific targets in interventions aimed at improving stress and anxiety problems in autistic adults.

## 1. Introduction

Autistic adults exhibit core and comorbid features that can disrupt daily functioning and lead to significant levels of psychological distress [1,2]. The risk of developing comorbid mental health conditions is high among autistic adults [3,4], manifesting as anxiety [5,6,7] and stress [8,9,10]. High self-perceived lifetime stress has been reported among autistic adults [8,11]. In fact, previous research suggests rates of current and lifetime anxiety for autistic adults to be between 27% and 42% for any anxiety disorder [6].

Several studies have explored the underlying mechanisms involved in the development and maintenance of stress and anxiety among autistic adults. Significant and consistent associations have been found between high levels of stress/anxiety and two specific autism spectrum disorder (ASD) characteristics: restricted repetitive behaviors (RRBs) and sensory features [12,13,14,15,16,17,18]. According to the literature, there is also a substantial relationship between RRBs and sensory features in autism [19,20,21,22]. To explain these relationships, research has proposed that RRBs may function as a mechanism that attempts to correct or alleviate arousal imbalances by increasing stimulation when the individual is under-aroused and by reducing stimulation when the individual is over-aroused [16,23].

Regarding the cognitive aspects that may explain variations in stress and anxiety levels in autistic adults, intolerance of uncertainty is one of the main variables explored and has been found to play a relevant role [24,25,26,27], but other cognitive processes such as worry, which is also related with psychological distress [28,29], have not been studied enough in autistic people. Given that cognitive processes seem to underlie the relationship between ASD features and psychological distress, further exploration in adult autistic populations is necessary to describe their possible role in clinical interventions.

In the current study, we analyzed the relationship between autism characteristics (sensory sensitivities and repetitive behaviors) and an autistic adult’s psychological distress (anxiety and stress), considering two mediating variables: worry and intolerance of uncertainty.

## 2. Outcome Variables: Anxiety and Perceived Stress

Anxiety disorders refer to various symptoms, such as restlessness, feeling on edge, being easily fatigued, difficulty concentrating, irritability, muscle tension, and sleep disturbances [30]. Kerns and Kendall found that DSM-specified anxiety disorders are prevalent and significantly more common in ASD (52%) compared with typical development (8%) [5]. Similarly, a meta-analysis showed that autistic adolescents tend to have higher anxiety levels compared to clinically referred children, and this difference increases when children with ASD have higher IQs and get older [31]. One possible explanation provided by the authors is that, with higher intellectual functioning, these individuals are more aware of their difficulties in adapting to the demands of the environment, which causes more anxiety and stress.

There is substantially less literature regarding stress in autistic people when compared with anxiety; however, researchers have recently begun conducting more studies including this variable. Perceived stress is defined as “the degree to which situations in one’s life are appraised as stressful” [32] (p. 385). Greater perceived stress is associated with less independence in activities of daily living and poorer subjective quality of life across all domains in adults with autism [10]. Adults with autism usually report significantly higher subjective stress and poorer ability to cope with stress in everyday life, as compared to typical adults [8,9,10,11]. Higher levels of autistic traits have been linked to greater perceived stress in both autistic and non-autistic individuals [12]. Research on ‘autistic burnout’—chronic exhaustion caused by the accumulation of life stressors that overwhelm coping capacities—further underscores this, showing that autistic individuals experience heightened stress and frequent encounters with stressors [33,34]. In addition, a study found that stress correlated with increased levels of anxiety in autistic adults, suggesting that the more anxious autistic individuals are, the less likely they are to be able to cope with the demands of the context, resulting in the perception of higher levels of stress [35].

## 3. Predictor Variables: Restrictive and Repetitive Behavior and Sensory Sensitivities

RRBs have been identified as a core feature of autism since the disorder was first described [36] and often manifest as an obsessive desire for sameness, verbal and motor rituals, obsessive questioning, and rigid adherence to routine [37,38]. However, different classifications and subtypes of RRBs can be found in the literature, depending on the measuring instrument used [39]. One of the more accepted conceptualizations suggests two categories: repetitive sensory and motor behaviors and insistence on sameness [40,41,42]. In a longitudinal study, Richler et al. analyzed the trajectories of sub-types of RRBs, finding that repetitive sensory and motor scores remained relatively high over time, indicating consistent severity, whereas insistence on sameness scores started low and increased over time [43]. RRBs have been shown as a relevant predictor of anxiety in children and adults with autism [13,15].

Atypical behavioral responses to sensory stimuli have been commonly observed in autistic people, and significant differences were found when they were compared with typical individuals [44,45]. Since 2013, hyper- or hypo-reactivity to sensory input or unusual interest in sensory aspects of the environment is considered a key diagnostic criterion for ASD [30]. Consequently, in recent years, a large body of research has focused on examining the difficulties in sensory processing and sensory subtypes in ASD [46,47,48,49,50,51,52]. Sensory sensitivities have been associated with anxiety in autistic adults [14,18]. In relation to the possible bidirectionality of this relationship, the results of an examination of the perceived causal relationships between sensory reactivity differences and anxiety in 246 autistic adults are particularly interesting. The study found that overall sensory hyper-reactivity, and visual, auditory, and olfactory hyper-reactivity were perceived as significantly more of a cause of anxiety than an effect, while overall sensory seeking, as well as tactile and vestibular seeking, were perceived as significantly more of an effect of anxiety than a cause [18].

Recent research has highlighted the intricate nature of the relationship between sensory features and RRBs [20,53], as well as their association with psychological distress in autism [38]. Studies have shown that high levels of hyperresponsive behaviors and sensory seeking are linked to ritualistic/sameness behaviors [19], both of which are significantly associated with internalizing symptoms [21]. In addition, Moore et al. found that intolerance of uncertainty and anxiety may act as mediators in the relationship between sensory processing and RRBs, suggesting significant roles in the interplay of these variables [54]. Researchers [38] identified a stress cycle where existing stress heightened sensory sensitivities, which in turn triggered more stress and diminished the ability to cope with additional stressors. While the self-regulatory benefits of RRBs on emotion, cognition, and self-fulfillment could help alleviate stress, this was obstructed by autistic individuals’ awareness of the stigma surrounding these behaviors, leading them to mask, which added further stress.

## 4. Mediating Variables: Worry and Intolerance of Uncertainty

Intolerance of uncertainty is regarded as a transdiagnostic construct characterized by an overevaluation of predictability and a tendency to become overwhelmed by the unexpected or the unknown [55,56]. In the general population, it is recognized as a dispositional risk factor in the development of anxiety disorders. The relationship between intolerance of uncertainty and anxiety in adults is robust [57]. Indeed, psychological interventions focused on increasing tolerance to uncertainty have demonstrated effectiveness in the reduction of anxiety in neurotypical populations [58].

Intolerance of uncertainty is an important factor to be considered in the conceptualization and management of elevated rates of anxiety for adults on the autism spectrum [59]. Autistic individuals demonstrate a greater intolerance of uncertainty compared to the non-autistic population [26,60]. One of the first studies analyzing this construct among autistic individuals found that when intolerance of uncertainty was controlled for, the variance in anxiety explained by the diagnosis was no longer significant, suggesting that intolerance of uncertainty might mediate the association between autism and anxiety [60].

Recent studies have identified intolerance of uncertainty as a significant mediator between autistic traits and anxiety [61,62], particularly in the relationship between sensory sensitivities and anxiety, as well as between anxiety and insistence on sameness behaviors [25]. However, the mediating role of intolerance of uncertainty between autism traits and stress remains unexplored, highlighting the need to understand the potential relationships among these factors.

In the general population, high correlations have been found between intolerance of uncertainty and worry [56,63], as well as with symptoms of anxiety [64] and depression [65]. High levels of worry seem to exacerbate the relationships between intolerance of uncertainty and problems with anxiety and depression [66].

Worry is a core and defining feature of anxiety and is believed to be a common mechanism contributing to different psychiatric conditions [67]. Worry refers to a cognitive tendency to ruminate on problems and the difficulty of putting them aside [68]. Excessive worry may be associated with difficulty differentiating nonthreatening from threatening cues and a tendency to respond inappropriately to nonthreatening cues, which interferes with adaptive behaviors, thereby maintaining chronic anxiety symptoms [29]. To a certain degree, worry can be adaptive, but it becomes pathological when it turns into frequent repetitive and negative thoughts about future peril that are very difficult for the individual to control [68,69]. This construct has been shown to be highly verbal, cognitive, and abstract in nature [70] and could resemble cognitive rather than somatic anxiety. Although it is a construct originally considered central to generalized anxiety disorder (GAD), its importance has been identified in other anxiety disorders, coming to be considered as an independent transdiagnostic construct [71].

In non-clinical populations, worry is associated with poorer mental health outcomes [72] and a tendency to exhibit prolonged physiological activation to stress [28]. Worry is positively associated with negative affect [73] and negatively related to well-being [74]. A study carried out on a non-clinical sample found that frequent aversive sensory experiences mediated the relationship between autistic traits and worry [75]. Interventions that treat excessive levels of recurrent worry among neurotypical populations have observed significant effects on anxiety and depression symptoms [76].

However, research into the role of worry in autistic people is scarce. In a recent study, autistic adults demonstrated clinically significant levels of worry, which were substantially higher than those in adults without autism. Autistic adults described worry as a cycle of negative thoughts impacting their participation in daily activities, sleep, and mental health [77].

For all these reasons, it is considered essential to study the role of these cognitive variables (worry and intolerance of uncertainty) in the relationships between autistic traits and psychological distress, using a complex model that allows for the exploration of their potential mediating role. This model should consider RRBs and sensory activities as predictor variables and both anxiety and stress as outcome variables.

## 5. Current Study

**Figure 1:**
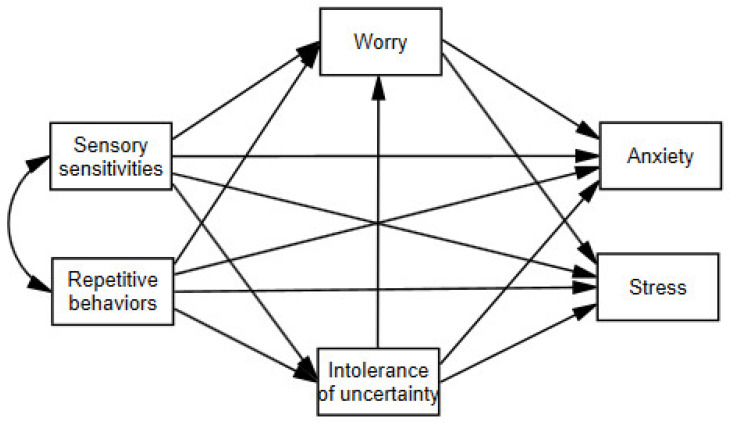
Proposed mediation model.

The aim of the current study was to analyze the relationships between the characteristics of ASD (sensory sensitivities and repetitive behaviors) and the levels of somatic anxiety and perceived stress, exploring the possible mediating role of two cognitive factors, namely, worry and intolerance of uncertainty (Figure 1). We predicted the following relationships: (1) Direct relationships of sensory sensitivities and repetitive behaviors with both measures of psychological distress (i.e., anxiety and stress); (2) Positive relationships of sensory sensitivities and repetitive behaviors with worry and intolerance of uncertainty; (3) Positive relationships of worry and intolerance of uncertainty with both measures of psychological distress; (4) A positive association between intolerance of uncertainty and worry; (5) Mediating roles of worry and intolerance of uncertainty in the relationships of sensory sensitivities and repetitive behaviors with both measures of psychological distress.

## 6. Methods

### 6.1. Participants

A total of 151 autistic individuals participated in the study. Of these, 112 (74.2%) were female and 35 (23.2%) were male. The participants’ ages ranged from 15 to 60 years, with only 5 cases between 15 and 17 years. The mean age was 35.15 years (SD = 11.44). In terms of marital status, 43.7% were single, 39.1% had a stable partner or were married, and 11.3% were divorced. Regarding educational level, there was a similar percentage of participants with primary and secondary education (48.2%) compared to those with a bachelor, master, or doctoral degree (51.8%). The average time since diagnosis was 5.51 years (SD = 5.43). The knowledge about the diagnosis of the participants comes from the information they themselves provided in the questionnaire. Most of the participants reported a diagnosis of Asperger syndrome (63.6%) or Level 1 ASD (27.1%). Seven participants who did not report formal diagnosis and scored lower than 26 on the Autism Spectrum Quotient (AQ) scale were excluded from the study. Only 12 participants who did not report formal diagnosis remained in the sample as they scored above 26 on the AQ scale. This resulted in a final sample size of 144 participants.

### 6.2. Instruments

For the evaluation of the study variables, Spanish self-reported instruments validated for adults were used. For the scales without a previous Spanish adaptation (Autism Spectrum Quotient and Repetitive Behaviors Questionnaire-2 Adult), an ad hoc back-translation process was conducted to ensure translation accuracy.

Sociodemographic questionnaire. A sociodemographic questionnaire was designed and included in the current study. The questionnaire contained questions considered of potential relevance for the description of the sample, such as gender, age, type of diagnosis, age of diagnosis, place of residence, marital status, educational level, employment status, and number of people living in the household of the participants.

Autism Spectrum Quotient (AQ). The AQ is a non-diagnostic, self-reported questionnaire to assess autistic symptomatology in adults [78,79]. It has 50 items, with a Likert-type structure with four options, indicating the degree of agreement or disagreement, with no neutral options. It is scored in a binary way, scoring one point for agreement on direct items and one point for disagreement on inverse items (regardless of the grade indicated). The total score results from a simple sum, providing a range of scores from 0 to 50. The usefulness of this instrument in identifying the extended autism phenotype has been suggested [80]. It can discriminate between patients with and without a diagnosis of Asperger syndrome [81], making it useful as a screening tool, with a currently accepted cutoff score of 26 (originally 32). For the present study, the global score was only used to ensure the exclusion of participants who reported a lack of formal diagnosis at the time of the study. Austin [82] reported a Cronbach’s α coefficient of 0.82 for the test. In the present sample, α = 0.84 was obtained, which indicates good internal consistency.

Adult Sensory Questionnaire (ASQ) [83]. The ASQ was designed as a screening instrument for sensory defensiveness (i.e., a negative reaction associated with alterations in sensory processing). It was chosen for its brevity and simplicity as a self-reported instrument with a single total score. A version of the cross-cultural adaptation to Spanish [84] was used. It has 26 items, with two response options (true-false). The range of total scores varies between 0 and 26, with Kinnealey and Oliver [83] suggesting that scores of 6 to 10 indicate moderate sensory defensiveness and scores above 10 indicate definite sensory defensiveness. Cronbach’s α in this study was 0.79, which can be considered good.

Repetitive Behaviors Questionnaire-2 Adult (RBQ-2A) [85]. The RBQ-2A is an adaptation of the RBQ-2 [86]. It is one of the few self-reported tools aimed at adults that specifically collects information on restricted and repetitive behaviors. Its psychometric characteristics have been subsequently analyzed by Barret et al. [87], along with its distribution into subfactors, finding one focused on repetitive motor and sensory behaviors (repetitive motor behaviors, or RMB) and a second one focused on insistence on similarity or invariability (insistence on sameness, IS). The questionnaire has been shown to have positive correlations with the AQ and good internal consistency (α = 0.73 and α = 0.87 in older participants). It has 20 questions about the degree of occurrence or severity of restricted and repetitive behaviors. For the present study, the total score was used. s. The version used in this study had good internal consistency (α = 0.84).

Intolerance of Uncertainty Scale (IUS). An abbreviated version of the IUS (IUS-12) [88] was used in the current study. This version has been shown to have good psychometric properties like the original version of 27 items [89]. This 12-item version has shown a high correlation with the 27-item version (r = 0.96) and high internal consistency (α = 0.91). The scale is made up of 12 items with descriptions, and participants must indicate to what degree they seem to be characteristic of themselves on a five-point Likert scale. Scores range from 12 to 60 points. The short version presents items identical to those of the complete version, but because no translated version was available, it was adapted ad hoc from the IUS version in Spanish and translated by León et al. [90]. In this study, a high internal consistency of α = 0.89 was obtained.

Penn State Worry Questionnaire (PSWQ). The PSWQ [91] is considered a reference in the measurement of worries, their occurrence, intrusiveness, and generalization. For this study, the Spanish translation was used [92]. The PSWQ is a self-reported questionnaire of 16 items on a five-point Likert scale, with a total score that varies between 16 and 80 points. Multiple cutoff points for the total score have been suggested for various clinical disorders. Three ranges were proposed: low concern (16–39), moderate to high concern (40–54), and high concern of a clinical nature (55–80) [93]. The adapted questionnaire has good convergent and divergent validity, as well as good internal consistency (α = 0.95). Cronbach’s alpha coefficient in the present work was quite high (α = 0.95).

Perceived Stress Scale (PSS). The Spanish adaptation of the PSS [94], originally developed by Cohen et al. [32], was used. It consists of 14 items on a five-point Likert-type scale where questions are asked about the frequency with which thoughts or feelings appear that may have caused stress (0 = never and 4 = very often). Total scores range from 0 to 56, with higher scores indicating a higher degree of stress. The internal consistency of the scale was acceptable (α = 0.77).

Beck Anxiety Inventory (BAI) [95]. The BAI was chosen for its brevity and usefulness in detecting symptoms of generalized anxiety. In the current study, the Spanish adaptation by Sanz and Navarro was used [96]. It is a self-reported measure with 21 items that represent possible anxiety symptoms. The participant must indicate, on a four-point Likert scale, the degree of trouble that each symptom has produced during the previous week, from *not at all troublesome* to *severely troublesome*. Each response is scored from 0 to 3, and the direct score is added to obtain a total that goes from 0 to 63. Minimal anxiety ranges from 0 to 7, mild anxiety from 8 to 15, moderate anxiety from 16 to 25, and severe anxiety from 26 to 63 [97]. Given its composition and the fact that most of the items in the inventory refer to physiological characteristics, the scores are considered to reflect somatic anxiety or simply anxiety. The original inventory has high internal consistency (α = 0.92) and test-retest reliability of r = 0.75 [95]. The reliability calculation yielded a Cronbach’s α of 0.95 for the sample collected, indicating very good internal consistency.

### 6.3. Procedure

Data collection was conducted online via the https://www.smartsurvey.co.uk/, accessed on platform. Representatives from various organizations that work with autistic people were contacted via a letter explaining the study’s objectives and requesting that they disseminate it among their users. Among the associations that agreed to participate are *Fundación Ángel Rivière*, *CEPAMA* (*Committee for the Promotion and Support of Autistic Women*), *Confederación Autismo Andalucía*, *Asociación BATA*, and *Autismo Burgos*. The professionals contacted autistic adults from their association and provided them with booklets or sent the link to the online survey to those who chose to participate. Additionally, a snowball sampling method was conducted. Autistic adults who were highly active on the social media platform Twitter were contacted and asked to share the study with their contacts. Participants in the online questionnaire received information about the study’s objectives and their right to withdraw at any time. They were assured of the anonymity and confidentiality of their responses. Only those who provided explicit consent were allowed to proceed with the questionnaire. The study adhered to the principles outlined in the Declaration of Helsinki.

### 6.4. Data Analyses

We evaluated multivariate normality using Mardia’s multivariate kurtosis coefficient [98], which considers a critical ratio of kurtosis < 10 to indicate multivariate normality [99]. Results revealed a Mardia’s multivariate kurtosis coefficient of 8.06, indicating the presence of multivariate normality. To examine the relationships between the variables measured, descriptive statistics and Pearson’s correlation coefficients were examined via IBM SPSS Statistics 27. Next, AMOS 27 was used for path analysis with maximum likelihood estimation to test the hypothesized mediating effects and simultaneously explain the interaction between the main variables [100]. To evaluate the model’s adequacy, several indices were used, as suggested by Kline [101]. For an acceptable fit, χ^2^/df < 3, CFI > 0.90, NFI > 0.90, and RMSEA < 0.08 were considered suitable criteria. For an excellent fit, χ^2^/df < 2, CFI > 0.95, NFI > 0.95, and RMSEA < 0.06 were considered indicators of a well-fitted model. To evaluate the mediator effects, a bootstrap procedure with 5000 resamples and a 95% bias-corrected confidence interval was conducted. If the confidence interval between the lower and upper bounds did not contain zero, the effect was considered statistically significant at *p* < 0.05.

## 7. Results

### 7.1. Preliminary Analyses

Descriptive statistics and bivariate correlations among all the observed variables are presented in Table 1. A high and significant direct linear relationship was observed among all variables in the study.

Overall, study participants showed clear sensory defensiveness (i.e., a negative reaction associated with alterations in sensory processing) and high clinical concern. The average score on the ASQ was 17.83, which exceeds the cutoff point of 10 established by Kinnealey and Olivier [83] and is indicative of definite sensory defensiveness. Likewise, the mean score on the PSWQ was 64.34, which falls between 55 and 80, the cutoff range suggested by Korte et al. [93] for high clinical concern. In addition, participants exhibited above-average scores on measures of repetitive behaviors [*t*(143) = 3.95, *p* < 0.01] and intolerance of uncertainty [*t*(143) = 11.77, *p* < 0.01], both of which exceeded the midpoint of their respective scales. However, their mean score for stress was below the midpoint of the scale [*t*(143) = 13.27, *p* < 0.01]. Finally, the participants’ mean score for anxiety was 27.22, which falls within the range of severe anxiety as defined by Beck and Steer [97].

### 7.2. Model Testing

**Figure 2:**
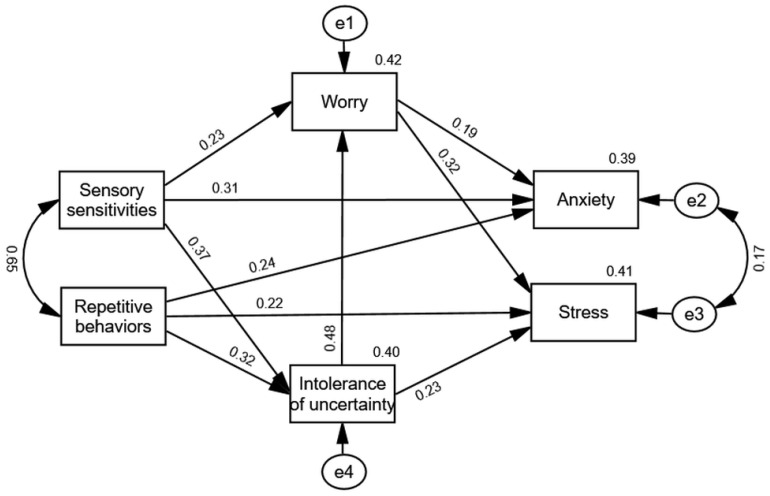
Standardized path coefficients among variables.

Except for RMSEA, model fit values tested through path analysis showed good fit (χ^2^ = 4.28, *p* < 0.05, CFI = 0.991, NFI = 0.989, RMSEA = 0.151, SRMR = 0.022). However, the direct effect between sensory sensitivities and stress was not found to be significant. This result supports the assumption of complete mediation, and we, therefore, excluded this insignificant direct effect. The same applied to the direct effect between repetitive behaviors and worry and between intolerance of uncertainty and anxiety, both of which were removed due to insignificance. The final model (see Figure 2) showed a good fit across all indicators (χ^2^ = 5.65, *p* = 0.13 ns, CFI = 0.993, NFI = 0.985, RMSEA = 0.079, SRMR = 0.025).

To examine the mediating effect of each variable, the paths from predictors to the other mediator were eliminated, so there was only one mediator at a time. The summary of direct and indirect effects is provided in Table 2.

As Figure 2 shows, sensory sensitivities predict worry (β = 0.23, *p* = 0.003), which in turn predicts the level of anxiety (β = 0.19, *p* = 0.014) and the level of stress (β = 0.40, *p* < 0.001). The variable worry plays a mediating role in the relationship between sensory sensitivities and both criterion variables (anxiety and stress). In the first case, there is partial mediation since the direct relationship between sensory sensitivities and anxiety decreased but remained significant when the mediator variable was introduced. In the second case, there is total mediation since the direct relationship between sensory sensitivities and stress dropped to a non-significant value when worry was introduced into the model. Additionally, the relationships between both predictors (sensory sensitivities, repetitive behaviors) and stress were mediated by intolerance of uncertainty, with total mediation in the case of sensory sensitivities and partial mediation in the other case (see Table 2). Furthermore, sensory sensitivities and repetitive behaviors are predictors of intolerance of uncertainty, which subsequently predicts the level of worry. Intolerance of uncertainty acts as a mediator between sensory sensitivities and worry (partial mediation), as well as between repetitive behaviors and worry (total mediation).

## 8. Discussion

The current study aimed to investigate, in a sample of autistic adults, how two features of ASD (sensory sensitivities and repetitive behaviors) relate to levels of anxiety and stress while examining whether two cognitive factors (worry and intolerance of uncertainty) mediate these relationships.

Overall, study participants showed clear sensory defensiveness, high clinical concern, and severe anxiety. In addition, participants had above-average scores on measures of repetitive behaviors and intolerance of uncertainty. However, their mean score for stress was below the midpoint of the scale. Bivariate correlations showed there was a high and significant direct linear relationship among all study variables. These findings are consistent with previous research that has demonstrated a strong relationship between core ASD features, such as sensory sensitivities and repetitive behaviors, and elevated levels of anxiety and stress in autistic adults [12,13,15].

Data analyses found that the hypothesized model fit well. The two autism traits studied (sensory sensitivities and repetitive behaviors) were shown to be predictors of anxiety and stress, although with some differences in the pattern of relationships. Repetitive behaviors showed a direct relationship with stress and anxiety, while sensory sensitivities only showed a direct relationship with anxiety. Furthermore, sensory sensitivities were found to predict worry, which in turn predicted anxiety and stress levels. The variable worry mediated the relationship between sensory sensitivities, anxiety, and stress levels. The relationships between sensory sensitivities and stress, as well as between repetitive behaviors and stress, were also mediated by intolerance of uncertainty. Additionally, intolerance of uncertainty acted as a partial mediator between sensory sensitivities and worry and as a total mediator between repetitive behaviors and worry. 

The mediating roles of intolerance of uncertainty and worry align with other author’s findings, which emphasize the importance of cognitive processes in predicting psychological distress among autistic adults [27]. This is also consistent with earlier studies that identified intolerance of uncertainty as a core cognitive feature exacerbating anxiety in autism [26,60]. Notably, our results extend the existing knowledge about the role of intolerance of uncertainty by demonstrating its mediating role in the relationships between sensory sensitivities and repetitive behaviors with stress, in addition to its previously documented association with anxiety [25].

As for worry, previous research in neurotypical populations shows that it plays a crucial role in maintaining anxiety [67,68], while the current study provides initial evidence that this construct is equally relevant for autistic individuals. In our study, the mediating role of worry highlights its significance as a cognitive process contributing to psychological distress. Although worry has not been extensively studied in autistic populations, our findings are aligned with Black et al. [77], who found that autistic adults experience clinically significant levels of worry, which can impact their daily functioning and exacerbate their anxiety and stress. 

Additionally, these findings underscore the importance of addressing both sensory processing difficulties and cognitive factors, such as worry and intolerance of uncertainty, in interventions aimed at reducing psychological distress in autistic adults. Further research is needed to explore these mechanisms in more depth, particularly in relation to clinical interventions.

In sum, the results are consistent with previous evidence showing that autistic adults who exhibit higher levels of sensory sensitivities and repetitive behaviors also demonstrate higher levels of anxiety and stress [12,13,14,15,16,18], and they add to previous evidence regarding the relationship between worry and intolerance of uncertainty with psychological distress [24,25,26,27]. The tested model provides valuable information for a better understanding of the psychological mechanisms underlying this phenomenon. The resulting model not only identifies worry and intolerance of uncertainty as mediators of these relationships but also allows for the identification of the relationship between these cognitive factors as relevant elements of a complex explanatory model. 

In the clinical field, our findings have practical implications insofar as they point to worry and intolerance of uncertainty as possible specific targets for interventions aimed at improving anxiety and stress in autistic individuals. As previous research has proposed [102], autistic adults may require a more individualized and less manualized approach to the treatment of emotional disturbances, such as anxiety. It has been recommended that counseling services provide information about the process in advance and include questions about accessibility needs within their assessment [103,104]. Learning directly from autistic adults’ insights may also help to improve the delivery of mental health programs for autistic adults [105]. Our results are aligned with these studies as we highlight the importance of tailoring mental health interventions to meet the needs of autistic individuals, considering the impact that two cognitive factors—worry and intolerance to uncertainty—have on their well-being. For instance, anticipating intervention aspects for autistic adults, such as sessions’ structure and characteristics, could contribute to decreasing uncertainty and worry during the treatment process.

All in all, from a practical perspective, an updated framework for clinical providers should be further explored in future research in order to identify other critical variables that support the mental health needs and preferences of autistic people.

The current study has several limitations. Firstly, the knowledge about the diagnosis of the participants comes from the information they themselves provided in the questionnaire. Second, some measures include overlapping items (i.e., the RBQ [repetitive behavior] is described to include items measuring sensory sensitivities; the BAI [anxiety] includes items pertaining to worry). Third, the sampling method used was not probabilistic but convenience and accessibility sampling, which does not guarantee the representativeness of the sample, was, so the generalization of the findings should be made with caution. Finally, there are limitations of the proposed model itself and limitations related to the nature of the study. On the one hand, anxiety and stress are complex constructs that involve a wide range of factors. The proposed model only allows for testing the pattern between the relationships of some of these factors. On the other hand, although advanced statistical analysis techniques are used in this study to consider the direction of relationships in the model, the ex post facto and cross-sectional nature of the study do not allow for conclusions of causality to be drawn from the tested relationships, so the results should be interpreted with caution.

However, our study adds to the limited literature exploring the role of worry in the variations in stress and anxiety levels in autistic adults, using a complex model in which we have included other relevant cognitive variables such as intolerance of uncertainty. The proposed model and the described findings shed light on the underlying factors influencing stress and anxiety in autistic adults, which can be used to enhance the effectiveness of interventions aimed at improving psychological well-being. Our results suggest that excessive worry and intolerance of uncertainty can be relevant targets for clinical interventions aimed at reducing stress and anxiety while also considering the impact of sensory sensitivities and repetitive behaviors on these cognitive factors.

## 9. Conclusions

This study examined the complex interplay between sensory sensitivities, repetitive behaviors, anxiety, and stress in autistic adults, emphasizing the mediating roles of two cognitive factors: worry and intolerance of uncertainty. Our findings show that sensory sensitivities and repetitive behaviors predict both anxiety and stress. Specifically, sensory sensitivities are directly associated with anxiety, while repetitive behaviors are related to both anxiety and stress. The proposed mediation model reveals that worry mediates the relationship between sensory sensitivities and both anxiety and stress, while intolerance of uncertainty mediates the relationships between sensory sensitivities and stress, as well as between repetitive behaviors and stress. These findings are in line with existing literature while offering valuable new insights into the psychological mechanisms underlying distress in autistic adults. They highlight that both worry and intolerance of uncertainty are relevant cognitive factors that should be considered as clinical targets in the treatment of psychological distress in autistic adults.

## Figures and Tables

**Table 1:** Means (M), standard deviations (SD), and Pearson correlation coefficients among the variables in the study (*n* = 144).

	Score Range	M	SD	2	3	4	5	6
1. Sensory sensitivities	0–26	17.83	4.62	0.65 **	0.51 **	0.59 **	0.56 **	0.47 **
2. Repetitive behaviors	20–60	42.56	7.78		0.49 **	0.57 **	0.53 **	0.51 **
3. Worry	16–80	64.34	12.48			0.61 **	0.46 **	0.56 **
4. Intolerance of uncertainty	12–60	45.58	9.66				0.51 **	0.57 **
5. Anxiety	0–63	27.22	14.38					0.49 **
6. Stress	0–56	22.84	7.99					

** *p* < 0.01.

**Table 2:** Results of mediation analyses.

Mediational Analysis	Direct Beta Without Mediator	Direct Beta with Mediator	Indirect Beta [CI]
SS → W → A	0.38 **	0.31 **	0.100 * [0.016–0.181]
SS → W → S	0.24 **	0.06	0.221 ** [0.083–0.349]
SS → IU → S	0.24 **	0.06	0.141 ** [0.059–0.247]
SS → IU → W	0.34 **	0.23 **	0.180 ** [0.099–0.293]
RB → IU → S	0.34 **	0.22 **	0.122 ** [0.044–0.237]
RB → IU → W	0.27 **	0.12	0.156 ** [0.048–0.286]

Notes: SS = sensory sensitivities; RB = repetitive behaviors; W = worry; IU = intolerance of uncertainty; A = anxiety; S = stress. * *p* < 0.05; ** *p* < 0.01.

## Data Availability

The data presented in this study are available on request from the corresponding author due to privacy.
